# Diagnostic Value of Serum Biomarkers for Invasive Aspergillosis in Haematologic Patients

**DOI:** 10.3390/jof10090661

**Published:** 2024-09-20

**Authors:** Isabel Montesinos, Imane Saad Albichr, Elodie Collinge, Bénédicte Delaere, Te-Din Huang, Pierre Bogaerts, Corentin Deckers, Mai Hamouda, Patrick M. Honoré, Pierre Bulpa, Anne Sonet

**Affiliations:** 1 Laboratory Medicine—Microbiology, Centre Hospitalier Universitaire (CHU) Université Catholique de Louvain (UCL) Namur Site Godinne, 5530 Yvoir, Belgium; imane.saadalbichr@chuuclnamur.uclouvain.be (I.S.A.); te-din.huang@chuuclnamur.uclouvain.be (T.-D.H.); pierre.bogaerts@chuuclnamur.uclouvain.be (P.B.); corentin.deckers@uclouvain.be (C.D.); 2 Haematological Department, Centre Hospitalier Universitaire (CHU) Université Catholique de Louvain (UCL) Namur Site Godinne, 5530 Yvoir, Belgium; elodie.collinge@chuuclnamur.uclouvain.be (E.C.); anne.sonet@chuuclnamur.uclouvain.be (A.S.); 3 Infectiology Department, Centre Hospitalier Universitaire (CHU) Université Catholique de Louvain (UCL) Namur Site Godinne, 5530 Yvoir, Belgium; benedicte.delaere@chuuclnamur.uclouvain.be; 4 Pharmacy Faculty, Namur University, 5000 Namur, Belgium; mai.hamouda@student.unamur.be; 5 Intensive Care Unit, Centre Hospitalier Universitaire (CHU) Université Catholique de Louvain (UCL) Namur Site Godinne, 5530 Yvoir, Belgium; patrick.honore@chuuclnamur.uclouvain.be (P.M.H.); pierre.bulpa@chuuclnamur.uclouvain.be (P.B.)

**Keywords:** biomarkers, invasive aspergillosis, haematologic patients, MycoGENIE PCR, Wako Beta-D-Glucan, galactomannan Ag VirClia, Galactomannan Ag Platelia

## Abstract

Background: Invasive aspergillosis (IA) is a significant cause of morbidity and mortality in patients with haematological malignancies. Accurate diagnosis of IA is challenging due to non-specific symptoms and the impact of antifungal prophylaxis on biomarker sensitivity. Methods: This retrospective study evaluated the diagnostic performance of three serum biomarkers: *Aspergillus* Galactomannan Ag VirClia Monotest^®^ (VirClia), Wako β-D-Glucan Test^®^ (Wako BDG), and MycoGENIE Real-Time PCR^®^ (MycoGENIE PCR). True positives were defined as patients with proven or probable IA (*n* = 14), with a positive Platelia *Aspergillus* Antigen^®^ (Platelia) serving as a mycological criterion. True negatives were identified as patients with a positive Platelia assay but classified as non-probable IA (*n* = 10) and outpatients who consistently tested negative with the Platelia test throughout the study period (*n* = 20). Results: Most patients diagnosed with proven or probable IA were acute myeloid leukaemia or myelodysplastic syndrome patients receiving mould-active antifungal prophylaxis or treatment (71%). VirClia demonstrated high sensitivity (100%) for detecting IA, with a specificity of 83%. Wako BDG and MycoGENIE PCR showed lower sensitivities for IA (57% and 64%, respectively). MycoGENIE PCR detected *Aspergillus* spp. and Mucorales in two patients. Conclusions: Accurate diagnosis of IA remains challenging, especially in patients who have received mould-active antifungal treatment. VirClia showed comparable performance to Platelia, suggesting its potential for routine use. However, Wako BDG and MycoGENIE PCR results were less favourable in our study cohort. Nevertheless, MycoGENIE PCR detected two probable co-infections with *Aspergillus* spp. and *Mucorales*.

## 1. Introduction

Invasive aspergillosis (IA) is a severe and potentially life-threatening fungal infection predominantly affecting immunocompromised individuals, particularly those with haematological malignancies or undergoing haematopoietic stem cell transplantation. The early and accurate diagnosis of IA remains a significant clinical challenge, and serum biomarkers such as galactomannan, β-D-glucan, and molecular detection of *Aspergillus* spp. offer the promise of non-invasive, rapid, and sensitive detection of *Aspergillus* spp. infections, facilitating timely and targeted therapeutic interventions [1,2].

Since the late 1990s, galactomannan, a polysaccharide component of the *Aspergillus* spp. cell wall, has emerged as an important serum biomarker for the early detection of IA [3,4,5]. The Platelia *Aspergillus* EIA (enzyme immunoassay) from Bio-Rad Laboratories (Platelia) has been the most widely used and extensively studied galactomannan detection kit, effectively holding a hegemony in this diagnostic area for many years. However, this has been challenged by the development of new diagnostic tools and kits aimed at improving sensitivity, specificity, and accessibility. One example is the *Aspergillus* galactomannan Ag VirClia Monotest from Vircell (VirClia). This kit uses a chemiluminescence immunoassay (CLIA) that offers several advantages over Platelia, including a single automated test, robust performance, and reduced turnaround time [6,7].

In addition to galactomannan, other biomarkers such as β-D-glucan and *Aspergillus* spp. DNA detected by polymerase chain reaction (PCR) have proven helpful in diagnosing IA. β-D-glucan, a fungal cell wall component, can be detected in the serum of patients with invasive fungal infections, including aspergillosis. PCR for *Aspergillus* spp. DNA detection in blood samples is another promising diagnostic method. It offers high sensitivity and specificity, allowing for the rapid and early identification of *Aspergillus* spp. infections. PCR can detect fungal DNA even in low quantities, making it a valuable addition to the diagnostic arsenal for IA [2,8]. PCR, as well as galactomannan, are mycological criteria in the EORTC/MSGERC definitions of IA [9]

The objective of this study was to evaluate the performance of different biomarkers in haematologic patient serum, specifically the *Aspergillus* galactomannan Ag VirClia Monotest^®^, the Wako Beta-Glucan Test^®^, and the MycoGENIE Real-Time PCR^®^, in the diagnosis of IA. By comparing these assays, we aimed to determine their clinical utility, sensitivity, specificity, and potential integration into routine diagnostic workflows. By enhancing our understanding of these diagnostic tools, we hope to improve the management and outcomes of haematologic patients at high risk for this devastating infection.

## 2. Materials and Methods

### 2.1. Institutional Setting and Patient Cohort

This study was conducted at the University Haematology Department of CHU UCL Namur (Godinne), which specialises in treating haematological disorders, including haematopoietic stem cell transplant (HSCT). It is the principal haematology service centre in the Wallonia region. The study population comprised individuals diagnosed with various haematological malignancies and those undergoing HSCT. All patients were regularly monitored for IA as part of their routine clinical care. We selected haematological patients with a positive galactomannan test (Platelia) (*n* = 24) from March 2023 to May 2024. Of these, two patients had proven IA, 12 patients had probable IA, and ten patients had no evidence of IA. We also included 20 haematological patients followed in the outpatient clinic with no evidence of IA and negative Platelia tests during this period as negative controls. Clinical haematologists performed IA classification following the Revised and Updated EORTC/MSGERC criteria, using Platelia test as the mycological criterion [9]. We reviewed the medical records to extract demographic data, risk factors, and the most relevant clinical information. This study was approved by the institutional ethics committee, ensuring that all research activities adhered to the highest ethical standards (CE Mont-Godinne 201/2023).

### 2.2. Platelia Aspergillus Antigen

The Platelia *Aspergillus* Ag assay^®^ (Bio-Rad Laboratories, Hercules, CA, USA) (Platelia) was used to detect galactomannan antigens in the patient serum. This assay uses a sandwich enzyme-linked immunosorbent assay (ELISA) principle. Serum samples were collected from patients with suspected IA and stored at −20 °C until analysis. The assay was performed according to the manufacturer’s instructions. The results were interpreted based on the optical density (OD) readings, with a cut-off value determined according to the manufacturer’s guidelines (OD index of ≥0.5).

### 2.3. Aspergillus Galactomannan Ag VirClia Monotest

The *Aspergillus* galactomannan Ag VirClia Monotest^®^ (Vircell Microbiologists, Granada, Spain) (VirClia) was used to detect galactomannan antigens in the patient serum. This assay employs automated chemiluminescence technology. Serum samples were collected from patients with suspected IA and stored at −20 °C until analysis. The assay was performed according to the manufacturer’s instructions. Sera were pre-treated before testing in the VirClia Lotus random access instrument using monotest format strips. Results are expressed as index values: <0.16 negative; 0.16–0.20 equivocal; >0.20 positive.

### 2.4. Wako β-D-Glucan Test

The Wako β-D-glucan assay^®^ (Fujifilm Wako Chemicals, Richmond, VA, USA) (Wako BDG) was used to detect β-D-glucan levels in patient serum. This assay is based on a kinetic turbidimetric technique for the detection of fungal cell wall components. Serum samples were collected from patients at the time of suspected IA and stored at −20 °C until analysis. The assay was performed according to the manufacturer’s instructions. Results were interpreted on the basis of kinetic measurements, with a cut-off value of 7 pg/mL set according to the manufacturer’s guidelines.

### 2.5. MycoGENIE Real-Time PCR

The MycoGENIE Real-Time PCR assay^®^ (Ademtech, Pessac, France) (MycoGENIE PCR) was used to detect *Aspergillus* spp. DNA in the patient serum. This assay uses real-time polymerase chain reaction (PCR) technology to detect *Aspergillus* spp. and *Mucor* spp. Serum samples were collected from patients with suspected IA and stored at −20 °C until analysis. The DNA was extracted from 1 mL of serum using the ELITe InGenius SP 1000 kit in the ELITe InGenius automated system (ELITechGroup, Puteaux, France). MycoGENIE PCR was performed according to the manufacturer’s instructions using Biorad CFX96 thermocycler (Bio-Rad Laboratories, Hercules, CA, USA). Serum samples were considered negative if the cycle threshold (ct) was >45.

### 2.6. Statistical Analysis

We calculated the sensitivity and specificity to evaluate each biomarker’s diagnostic performance. True positives were defined as patients with proven or probable IA (*n* = 14), while the true negatives were patients with a positive galactomannan test but classified as non-probable IA and the 20 negative control patients. The following cut-off values were used for the respective tests: Platelia: ≥0.5, VirClia: ≥0.16 (both equivocal and positive results), Wako BDG: ≥7 pg/m, MycoGENIE PCR: ct < 45. Receiver operating characteristic (ROC) curves were generated for Platelia, VirClia, and Wako BDG to assess their diagnostic accuracy and determine the optimal cut-off values. The area under the curve (AUC), along with 95% confidence intervals, was calculated to compare the performance of the biomarkers. We calculated Spearman’s rank correlation coefficient to investigate the relationship between the two galactomannan tests, Platelia and VirClia, in our study sample. We considered a *p* value of <0.05 to be statistically significant. All statistical calculations were performed using MedCalc^®^ Statistical Software 23.0.2 (MedCalc Software Ltd., Ostend, Belgium; https://www.medcalc.org (accessed on 16 September 2024); 2024).

## 3. Results

### Patient Characteristics

During the study period, 24 patients with a positive Platelia test were identified. The demographic, underlying conditions and clinical characteristics of these patients are summarised in Table 1. In addition, 20 haematological patients followed in the outpatient clinic without any positive galactomannan tests during the study period and no evidence of IA were included as negative controls: 14 Acute Myeloid Leukemia (AML) patients, 4 Acute Lymphoblastic Leukemia (ALL) patients, and 2 with Myelodysplastic Syndrome (MDS) patients.

Table 2 provides an overview of the performance metrics, including sensitivity, specificity, and area under the curve (AUC) for the different biomarkers used in the study to diagnose IA in the haematological patients included in this study.

**Figure 1:**
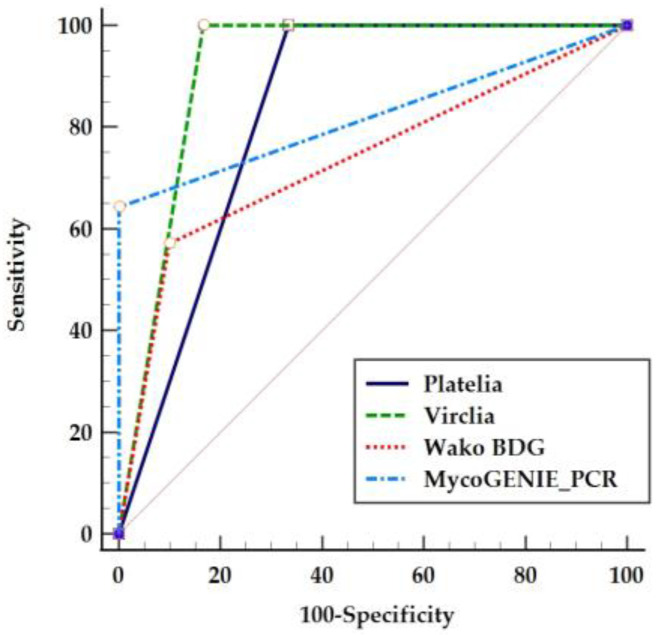
Receiver operating characteristic curves of biomarkers for the diagnosis of IA in haematological patients: Platelia *Aspergillus* Antigen^®^, *Aspergillus* Galactomannan Ag VirClia Monotest^®^, Wako β-D-Glucan Test^®^, and MycoGENIE Real-Time PCR^®^. The grey diagonal line represents the performance of a random classifier with no discriminative power.

We calculated the receiver operating characteristic (ROC) curves for VirClia, Wako BDG, and MycoGENIE PCR to assess their diagnostic accuracy and determine the optimal cut-off values (Figure 1). Pairwise comparison of the ROC curves indicates that while VirClia has a similar diagnostic performance to Platelia (difference between areas (DBA) = 0.00119; *p* = 0.97), both Platelia and VirClia show significantly better performance compared to Wako BDG (DBA = 0.174; *p* = 0.03 and DBA = 0.173; *p* = 0.01, respectively). MycoGENIE PCR did not show significantly different performance when compared to Platelia, VirClia, or Wako BDG (DBA = 0.114; *p* = 0.12, DBA = 0.113; *p* = 0.14, and DBA = 0.059; *p* = 0.64, respectively). The optimal cut-offs proposed by ROC curve calculation for VirClia, Wako BDG, and MycoGENIE PCR were index >0.13, >8.1 pg/mL, and ct < 40, respectively.

**Figure 2:**
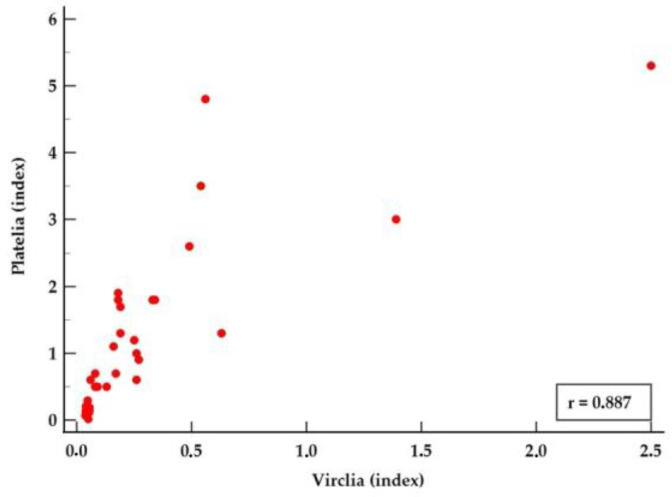
Scatter diagram of the correlation between Platelia *Aspergillus* Antigen^®^ and *Aspergillus* Galactomannan Ag VirClia Monotest^®^ results. R = Spearman’s coefficient of rank correlation (rho).

We found a correlation coefficient of 0.885 (95% CI: 0.80 to 0.93), indicating a strong and significant positive correlation between Platelia and VirClia galactomannan tests (*p* < 0.01). Figure 2 shows the scatter diagram of the correlation between Platelia and VirClia results.

During the study period, *Aspergillus* spp. and *Mucorales* were detected in two patients. The samples were sent to the Belgian Mycology Reference Center, where PCR confirmed the co-detection.

## 4. Discussion

In this study, we evaluated the diagnostic performance of three serum biomarkers in the detection of IA in a cohort of haematological patients: VirClia, Wako BDG, and MycoGENIE PCR. Most patients diagnosed with IA were AML or MDS patients (64%), underwent chemotherapy (86%), and had undergone AlloSCT (43% compared to 7% who underwent AutoSCT), as shown in other studies [10]. Additionally, most received mould-active antifungal prophylaxis or treatment (71%). Antifungal exposure can suppress fungal growth, leading to false-negative results in cultures and reduced sensitivity in biomarker assays, thus hindering timely and accurate diagnosis [10,11,12,13]. Our findings indicate variable performances across the biomarkers, highlighting their strengths and limitations in clinical practice.

VirClia demonstrated high sensitivity for the detection of IA, in line with previous studies highlighting the utility of galactomannan assays in diagnosing IA among immunocompromised patients [14,15]. However, the sensitivity reported in this study may be artificially elevated due to selection bias, as we focused on patients with pre-existing positive galactomannan results. This criterion can skew the data by excluding patients who may have IA but present with negative galactomannan tests. This issue is particularly pertinent in our cohort, where a significant proportion of patients were receiving mould-active antifungal prophylaxis or treatment at the time of testing. In these patients, the serum galactomannan test has reduced accuracy and is not recommended for screening in preemptive strategies but rather for a driven diagnostic approach [10,11]. ROC curve analysis and pairwise comparisons reveal that Platelia and VirClia have comparable diagnostic performance, suggesting that both tests could be used interchangeably in clinical practice. It should be noted that in this study, VirClia equivocal results (index > 0.16) were included as positive for performance analysis, and the sensitivity could be lower if only positive results (index > 0.2) were considered. Additionally, the optimal cut-off suggested by the ROC curve calculation (index > 0.13) indicates that the manufacturer may consider lowering the cut-off value to improve accuracy. VirClia demonstrated higher specificity (83% compared to 67% for Platelia) than expected in a haematological population exposed to antifungal agents. This higher specificity is critical in this patient population as it reduces the likelihood of false positives, which can lead to unnecessary antifungal treatment and potential side effects. The superior specificity of VirClia compared to Platelia may be due to differences in assay design, including antigen selection and detection methods. Clinically, this suggests that VirClia may be more reliable in ruling out IA in non-IA patients, potentially reducing the need for further invasive diagnostic procedures. The results from Platelia and VirClia results also showed a strong correlation. Studies evaluating VirClia corroborate these findings, highlighting its simplicity and automated mono-test format, which reduces turnaround times and enhances user-friendliness [6,7,16]. However, the fact that VirClia did not improved the sensitivity over Platelia underscores the ongoing challenge of developing diagnostic methods that achieve both high sensitivity and maintain good specificity for IA. Despite the operational advantages of VirClia, the need for a diagnostic tool that can reliably combine these two characteristics remains crucial in clinical practice.

β-D-glucan (BDG) is a panfungal biomarker that has shown significant efficacy in diagnosing invasive fungal infections, particularly candidemia and pneumocystosis, due to its high negative predictive value (NPV). This makes it a valuable tool for ruling out fungal infections in patients with suspected invasive fungal infections (IFIs) [14,17,18,19]. However, its performance is less reliable for IA, especially in patients on antifungal prophylaxis or treatment [13]. Studies have highlighted the limitations of BDG testing in detecting IA, as BDG levels are often not as elevated in aspergillosis as in other fungal diseases, potentially leading to false negatives [20,21]. As expected, Wako BDG demonstrated significantly lower performance compared to the other tests analysed in our study. Therefore, while BDG testing remains valuable for ruling out fungal infections due to its high NPV, it should be used in conjunction with other diagnostic methods when IA is suspected.

Like the Wako BDG test, MycoGENIE PCR showed moderate sensitivity in our study. *Aspergillus* spp. PCR has recently been introduced as a mycological criterion for IA and has shown good performance in serum samples in several studies [9,22]. The impact of mould-active antifungal exposure on the performance of serum *Aspergillus* spp. PCR is a controversial issue [12,23]. A recent study suggested that, unlike galactomannan and BDG tests, the robustness of serum *Aspergillus* spp. PCR may not be affected by mould-active antifungal exposure. In the present study, we do not know how serum *Aspergillus* spp. PCR would perform in patients with suspected IA but negative galactomannan results (possible IA according to EORTC/MSGERC criteria), especially considering that antifungal exposure could lead to increased DNAemia at the onset of the infection. If so, serum *Aspergillus* spp. PCR would be an excellent test for screening or diagnosis of IA, allowing the identification of possible IA cases, that could then be reclassified as probable IA cases. However, the moderate sensitivity observed in this and other studies suggests the need for further evaluation of Aspergillus PCR as a diagnostic criterion in the EORTC/MSGERC guidelines. This finding contributes to the ongoing discussion among experts regarding the most appropriate biomarkers for the diagnosis of IA, particularly in patients receiving mould-active antifungal agents. [2,24,25,26]. Although MycoGENIE PCR has been less extensively evaluated for *Mucorales* detection, it has shown good performance in initial studies. It is particularly useful for detecting invasive mucormycosis, which can clinically resemble IA [27]. Serum *Mucorales* PCR is useful in the early diagnosis of invasive infections caused by these fungi [28]. In our study, MycoGENIE PCR detected probable co-infections with *Aspergillus* spp. and *Mucorales* in two patients, both of whom unfortunately died shortly afterwards. These findings could not be confirmed histologically as no autopsies were performed. This highlights the severity and rapid progression of fungal co-infections in this vulnerable population. The ability of MycoGENIE PCR to detect both pathogens underlines its value as a diagnostic tool in the management of complex fungal infections.

This study has some limitations that need to be considered. First, its retrospective nature and the selection of patients with pre-existing positive galactomannan tests introduce inherent biases in data collection and analysis. This selection bias could lead to the overestimation of the diagnostic accuracy of galactomannan testing, especially considering that sensitivity is reduced in patients on antifungal prophylaxis. Due to this method of patient selection, patients with possible IA were not included, leaving the performance of the biomarkers studied, particularly PCR, unknown for this group, as discussed above. Another limitation associated with the retrospective study design is the freezing and thawing of serum samples, which may have affected the performance of the tests. In addition, the number of patients with proven or probable IA in our cohort was limited, which may affect the generalisability of our findings.

## 5. Conclusions

Despite mould-active antifungal prophylaxis, an accurate diagnosis of IA remains challenging due to non-specific symptoms and the impact of antifungal treatment on biomarker sensitivity. Although Platelia is considered the gold standard for detecting galactomannan antigen, VirClia represents a viable alternative with comparable performance and can be used routinely in clinical practice. Larger prospective studies are necessary to validate and provide a more comprehensive evaluation of the performance of Wako BDG and MycoGENIE PCR in haematological populations with suspected IA.

## Figures and Tables

**Table 1:** Demographic, haematological underlying diseases, and clinical characteristics of patients with positive Platelia galactomannan in the period of the study.

	Proven/Probable IA ^1^ (*n* = 14)	No IA ^1^ (*n* = 10)
Demographics		
Age (median years, range)	66 (31–76)	66 (46–72)
Gender (male/female)	12/2	4/6
Haematologic underlying diseases		
AML^2^ (%)	7 (50%)	4 (40%)
MDS ^3^ (%)	2 (14)%	4 (40%)
NHL ^4^ (%)	2 (14)%	
CLL ^5^ (%)	1 (7%)	
ALL ^6^ (%)		1 (10%)
Others (%)	2 (14)%	1 (10%)
Risk factors and clinical characteristics		
AlloSCT ^7^	6 (43%)	10 (100%)
AutoSCT ^8^	1 (7%)	
Chemotherapy	12 (86%)	5 (50%)
Immunosuppressive therapy	7 (50%)	8 (80%)
GVDH ^9^	4 (28%)	5 (50%)
Febrile neutropenia	9 (64%)	1 (10%)
Antifungal exposure ^10^	10 (71%)	2 (20%)
Mortalities ^11^	9 (64%)	2 (20%)

^1^ EORTC/MSGERC definition of Invasive Aspergillosis, ^2^ Acute Myeloid Leukemia, ^3^ Myelodysplastic Syndrome, ^4^ Non-Hodgkin Lymphoma, ^5^ Chronic Lymphocytic Leukemia, ^6^ Acute Lymphoblastic Leukemia, ^7^ Allogenic Stem Cell Transplant, ^8^ Autologous Stem Cell Transplant, ^9^ Graft-Versus-Host Disease, ^10^ Mould-active antifungal prevention or treatment at the moment of positive galactomannan test, ^11^ 30-day mortality after positive galactomannan test.

**Table 2:** Sensitivities and specificities.

Biomarker	Cutoff Value	Sens ^1^ (%) (95% CI)	Spec ^2^ (%) (95% CI)	AUC ^3^ (95% CI)
VirClia ^4^	Index ≥ 0.16	100% (77–100)	83% (65–94)	0.92 (0.79–0.98)
Wako BDG ^5^	≥7 pg/mL	57% (29–82)	90% (73–98)	0.77 (0.58–0.88)
MycoGENIE PCR ^6^	ct < 45	64% (35–87)	100% (88–100)	0.82 (0.67–0.92)

^1^ Sensitivity, ^2^ Specificity, ^3^ Area Under the Curve, ^4^ *Aspergillus* Galactomannan Ag VirClia Monotest, ^5^ Wako β-D-Glucan Test, ^6^ MycoGENIE Real-Time PCR.

## Data Availability

The original contributions presented in the study are included in the article, further inquiries can be directed to the corresponding author.
